# Draft genome sequence of *Metabacillus indicus* strain EGFCL74 isolated from spontaneously fermented apple cider

**DOI:** 10.1128/mra.01023-23

**Published:** 2024-01-17

**Authors:** Eileen V. Garcia-Fuentes, Christopher A. Lopez

**Affiliations:** 1 Department of Biological Sciences, California State University Sacramento, Sacramento, California, USA; University of Maryland School of Medicine, Baltimore, Maryland, USA

**Keywords:** *Metabacillus indicus*, fermentation, cider

## Abstract

Here, we report the draft genome and annotations for *Metabacillus indicus* strain EGFCL74, a bacterium isolated from spontaneously fermented apples. This 4.10-Mb genome adds to the limited existing data on a potential spoilage organism in natural cider ferments.

## ANNOUNCEMENT

The Gram-positive bacterium *Metabacillus indicus* (previously *Bacillus indicus*) was initially cultured from arsenic-contaminated aquifer sand (1). The genera were separated based on phenotypic differences and six unique genes in *Metabacillus* (2). The yellow-orange colony pigment originates from carotenoids in the cell envelope (3) that contribute to interest in the potential probiotic application of the bacterium (4). Additionally, *M. indicus* contain industrially relevant pullulanases, starch-debranching enzymes necessary for saccharification (5). Other features of *M. indicus* have not been explored.

Here, we isolated *M. indicus* from a spontaneous apple cider ferment during a study on apple-associated microorganisms. Apples were obtained from the Almanor basin in the Northern Californian Sierra mountains (40.302395,–121.234672) at an elevation of 4,500 feet. The crushed and pressed juice, or must, was fermented at 20°C in a glass vessel for 6 months followed by 6 months maturation at 4°C in the same vessel. Post-maturation sediment was diluted in phosphate-buffered saline and plated onto brain heart infusion supplemented with yeast extract (BHIS) agar, followed by incubation at 26°C for 48 hours aerobically. Gram-stained cells from an orange-hued colony indicated Gram-positive bacilli. For whole-genome sequencing, the isolate was revived from storage at −80°C on BHIS agar at 26°C for 48 hours. Genomic DNA was recovered using the Monarch Genomic DNA Purification Kit (New England Biolabs) following the manufacturer’s instructions for Gram-positive bacteria.

The Nextera XT DNA Library Preparation Kit (Illumina) was used with 1 ng genomic DNA and an equivalent amount of Nextera XT fragmentation enzyme (Illumina). IDT unique dual indexes were added, followed by 12 cycles of PCR. The library was purified using AMpure magnetic Beads (Beckman Coulter) and then eluted with Qiagen EB buffer. The DNA library was quantified using a Qubit dsDNA HS Assay Kit and Qubit 4 fluorometer. CosmosID performed sequencing on an Illumina NovaSeq platform 2 × 150 bp, producing 2,012,946 reads.

Raw paired-end reads were trimmed and processed with BBDUK v39.01 (6) with a read quality trimming parameter of 22. Trimmed FASTQ files were uploaded to KBase (7) and assembled using SPAdes v3.15.3 (8) under the -careful parameter. The assembled genome has 4,095,815 bp and a *N*_50_ of 258,308. Completeness through CheckM v1.0.18 using the lineage_wf function (9) was 99.56% with 1.73% contamination.

**Fig 1:**
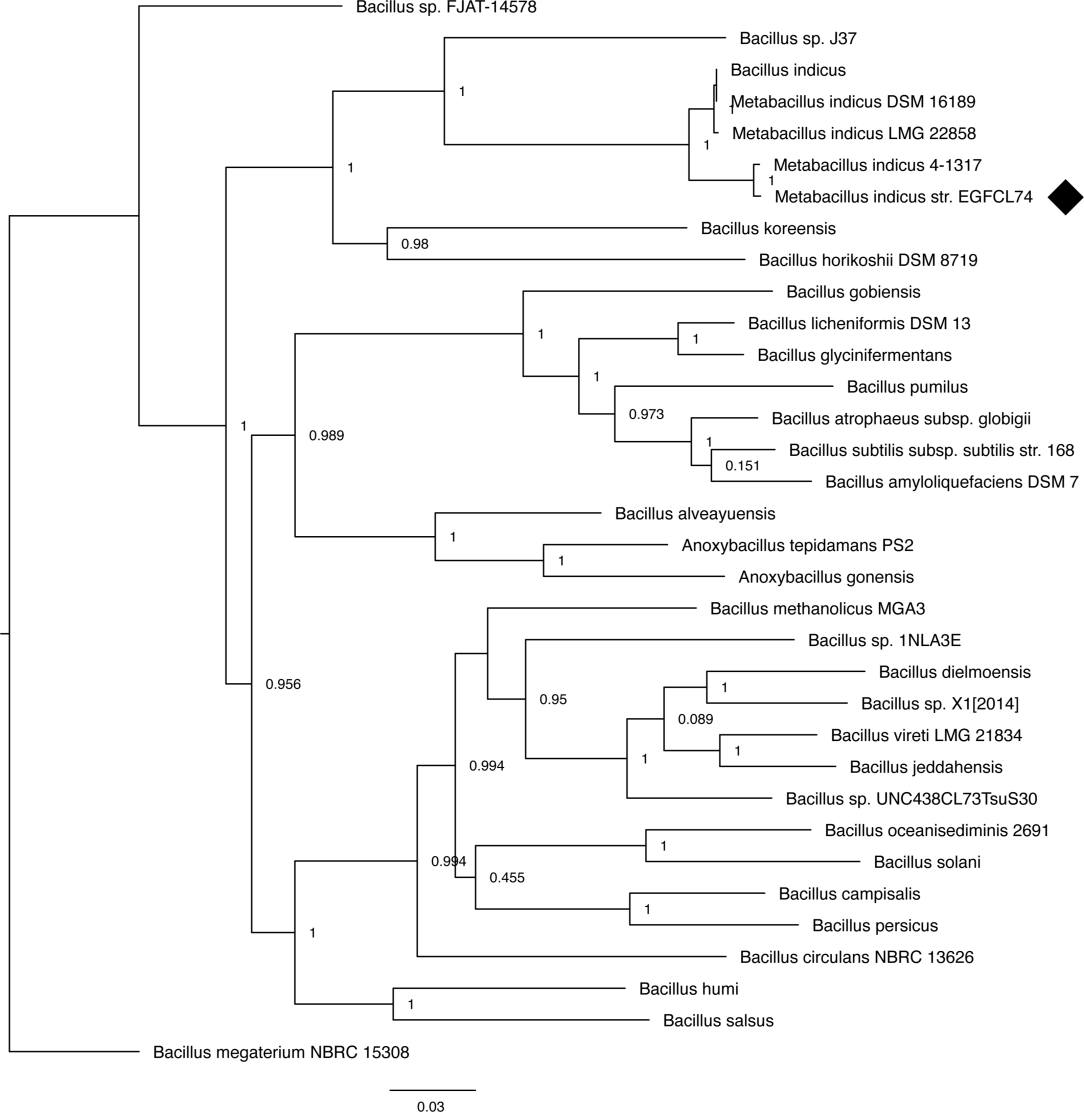
Evolutionary relationship of isolate to other sequenced genomes. The phylogenetic tree was constructed by estimating the maximum likelihood of phylogeny based on concatenated multiple sequence alignments of 49 core, universal genes defined by Clusters of Orthologous Groups in 30 closely related genomes plus 3 publicly available *Metabacillus* genomes. The initial tree was constructed in the Kbase “Insert_Genome_into_SpeciesTree” function v2.2.0 and FastTree2 v2.1.10 (10). The tree was edited with the apple-associated *Bacillus megaterium* as the outgroup using FigTree 1.4.4 (http://tree.bio.ed.ac.uk/software/figtree/).

The genome was annotated using RAStk v1.073 within KBase (https://doi.org/10.25982/163445.15/2223098). The genome clustered with *M. indicus* (Fig. 1) (10) and has a GC content of 44.60% with 4,389 predicted protein-coding genes over 34 contigs. Identity was confirmed by comparing average nucleotide identity (ANI) to available *M. indicus* genomes in FastANI v0.1.3, resulting in ANI values of 97.209% (strain 4-1317), 96.7094% (strain DSM16189), and 96.7856% (strain LMG22858). Percentages greater than 95% are considered the same species. All software were used under default parameters unless noted. To confirm strain uniqueness, six conserved *Metabacillus* gene sequences were compared through ClustalO v1.2.4 (Table 1). Sequence differences, particularly in *recQ*, support the novel strain designation of *Metabacillus indicus* strain EGFCL74.

**TABLE 1:** Comparison of DNA sequence similarity between isolate and other assembled *Metabacillus* genomes^*a*^

	DNA sequence similarity of six *Metabacillus* genes (% similarity to strain EGFCL74, gene length)					
	Fibronectin /fibrinogen-binding protein	Spore protease *yyaC*	DEAD/DEAH box helicase *cshB*	DEAD/DEAH box helicase *cshA*	ATP-dependent DNA helicase *recQ*	3-phosphoshikimate 1-carboxyvinyltransferase
EGFCL74 (this study)	100%, 1,704 bp	100%, 624 bp	100%, 1,317 bp	100%, 1,500 bp	100%, 2,118 bp	100%, 1,311 bp
4–1317 (GCA_947495645.1)	96.83%, 1,704 bp	96.47%, 624 bp	99.39%, 1,317 bp	99.53%, 1,503 bp	96.03%, 2,118 bp	96.95%, 1,311 bp
DSM 16189 (GCA_000709935.2)	96.95%, 1,704 bp	98.40%, 624 bp	99.39%, 1,317 bp	99.60%, 1,500 bp	89.94%, 2,118 bp	97.25%, 1,311 bp
LMG 22858 (GCA_000708755.2)	96.95%, 1,704 bp	98.40%, 624 bp	99.39%, 1,317 bp	99.60%, 1,503 bp	90.00%, 2,109 bp	97.33%, 1,311 bp

^*a*^ The table above compares the DNA sequences of six conserved *Metabacillus* genes (2) among the proposed strain (EGFCL74) to published *M. indicus* strains. Percentages indicate gene sequence similarity to strain EGFCL74. Gene size is displayed in base pairs (bp).

## Data Availability

The draft genome sequence of Metabacillus indicus was deposited into the Sequenced Read Archive (SRA) database under SRR26030092. Annotations and other data described here are available through KBase at https://doi.org/10.25982/163445.15/2223098. The assembled genome was also deposited in GenBank as GCA_033952665.1, where it was reannotated using the NCBI Prokaryotic Genome Annotation Pipeline.
